# The efficacy of a filtered handheld far-ultraviolet disinfection device for decontamination of high-touch surfaces in healthcare settings: a genomic bacterial analysis

**DOI:** 10.1017/ice.2025.10251

**Published:** 2025-10

**Authors:** Layale Yaghi, Roy F Chemaly, Rita Wilson-Dib, William C. Shropshire, Sherry Cantu, Micah Bhatti, Piyali Chatterjee, Chetan Jinadatha, Amy Spallone

**Affiliations:** 1 Department of Infectious Diseases, Infection Control and Employee Health, The University of Texas MD Anderson Cancer Center, Houston, TX, USA; 2 The University of Oklahoma College of Medicine, Oklahoma City, OK, USA; 3 Department of Infection Control, Chief Quality Office, The University of Texas MD Anderson Cancer Center, Houston, TX, USA; 4 Department of Laboratory Medicine, The University of Texas MD Anderson Cancer Center, Houston, TX, USA; 5 Department of Medical Education, College of Medicine, Texas A&M University, Bryan, TX, USA; 6 Department of Medicine, Central Texas Veterans Health Care System, Temple, TX, USA; 7 Department of Research, Central Texas Veterans Health Care System, Temple, TX, USA

## Abstract

**Background and objectives:**

Enhanced environmental disinfection is linked to reduced hospital-acquired infection rates. In this study, we aimed to evaluate the efficacy of an emerging disinfection technology, a filtered far-UV-C handheld (FFUHH) device, for reducing bacterial loads on high-touch surfaces in shared clinical workrooms, and to isolate, identify and characterize clinically significant environmental pathogens.

**Methods:**

We compared samples from high-touch items (dictation device, mouse, armchair, desk, and keyboard) before and after FFUHH treatment. Samples were collected weekly: contact plates for colony counts and swabs before and after intervention on standardized adjacent areas for each surface, respectively. The swabs were enriched and cultured on selective media to isolate pathogens. Environmental samples, as well as clinical samples collected from patients during the study period, were validated using MALDI-TOF and whole genome sequencing.

**Results:**

Among the 440 collected plates (220 before and 220 after treatment), the highest mean colony count pre-treatment was detected from armchairs, and the lowest from keyboards. The mean reduction of colony-forming units ranged 53% and 83% and was statistically significant (*P* < 0.05) across all surfaces except for the keyboard. We characterized multidrug-resistant *Staphylococcus epidermidis* ST5 and ST16 strains, a carbapenem-resistant *Acinetobacter baumannii,* and a *Klebsiella pneumoniae* genetically related to a clinical isolate with a rare sequence type not previously detected in our institution.

**Conclusion:**

The FFUHH effectively reduced the microbial burden on high-touch surfaces. It can offer an advantage for surface disinfection and an alternative to routinely used biocides.

## Introduction

The Centers for Disease Control and Prevention estimates that hospital-acquired infections (HAIs) occur in 1 in 31 hospitalized patients,^1^ with more than 1 million cases in the U.S. annually. The persistence of HAIs is attributable, in part, to the overuse of antimicrobial agents, the high prevalence of increasingly hard-to-treat multidrug-resistant organisms (MDROs) associated with HAIs, like methicillin-resistant *Staphylococcus aureus* (MRSA), vancomycin-resistant *Enterococcus* (VRE), and *Enterobacteriaceae*,^2^ and finally, suboptimal implementation of infection control practices.^3^

Approximately 55% of all HAIs are preventable through infection control measures.^4^ Pathogens can persist on dry surfaces for months or years,^5^ and contaminated high-touch surfaces have been linked to increased HAI risk,^6^ as have horizontal transmission from healthcare workers^7^ and resistance to standard surface disinfectants.^8^ This increases the likelihood of horizontal transfer of pathogens even when they are not in proximity to patients.^9^ However, evidence of a direct link of transmission from high-touch surfaces outside patients’ rooms to patients is lacking.^10^

Enhanced disinfection and cleaning thoroughness are linked to reduced HAI rates.^11^ The randomized REACH study highlighted that proper training of service workers and implementation of thorough cleaning protocols for frequently touched surfaces significantly reduced HAIs.^12^ This was corroborated by two systematic reviews that reported a decrease in patient infection with MDROs and/or HAI rates with different cleaning and disinfection strategies.^13,14^

Focus has been placed on emerging technologies, such as automated disinfection and environmental monitoring systems.^6^ The filtered Far UV-C handheld device (BeamClean^TM^ FFUHH, Freestyle Partners, USA) is a surface disinfecting portable device that emits ultraviolet (UV) light at 222 nm with germicidal capabilities at short contact times.^15,16^ This technology has also been proven safe in preclinical and clinical studies.^17,18^

In this study, we aimed to (i) evaluate the FFUHH’s efficacy in reducing the microbial burden on shared, high-touch surfaces in clinical workrooms, and (ii) isolate, identify and evaluate genetic relationship between recovered environmental pathogens and clinical isolates collected from patients in the same units, during the study period. We focused on 5 clinically significant pathogens: MRSA, VRE, *Pseudomonas (P.) aeruginosa*, *Klebsiella (K.) pneumoniae*, and *Escherichia (E.) coli*.

## Methods

### FFUHH device

**Figure 1.**
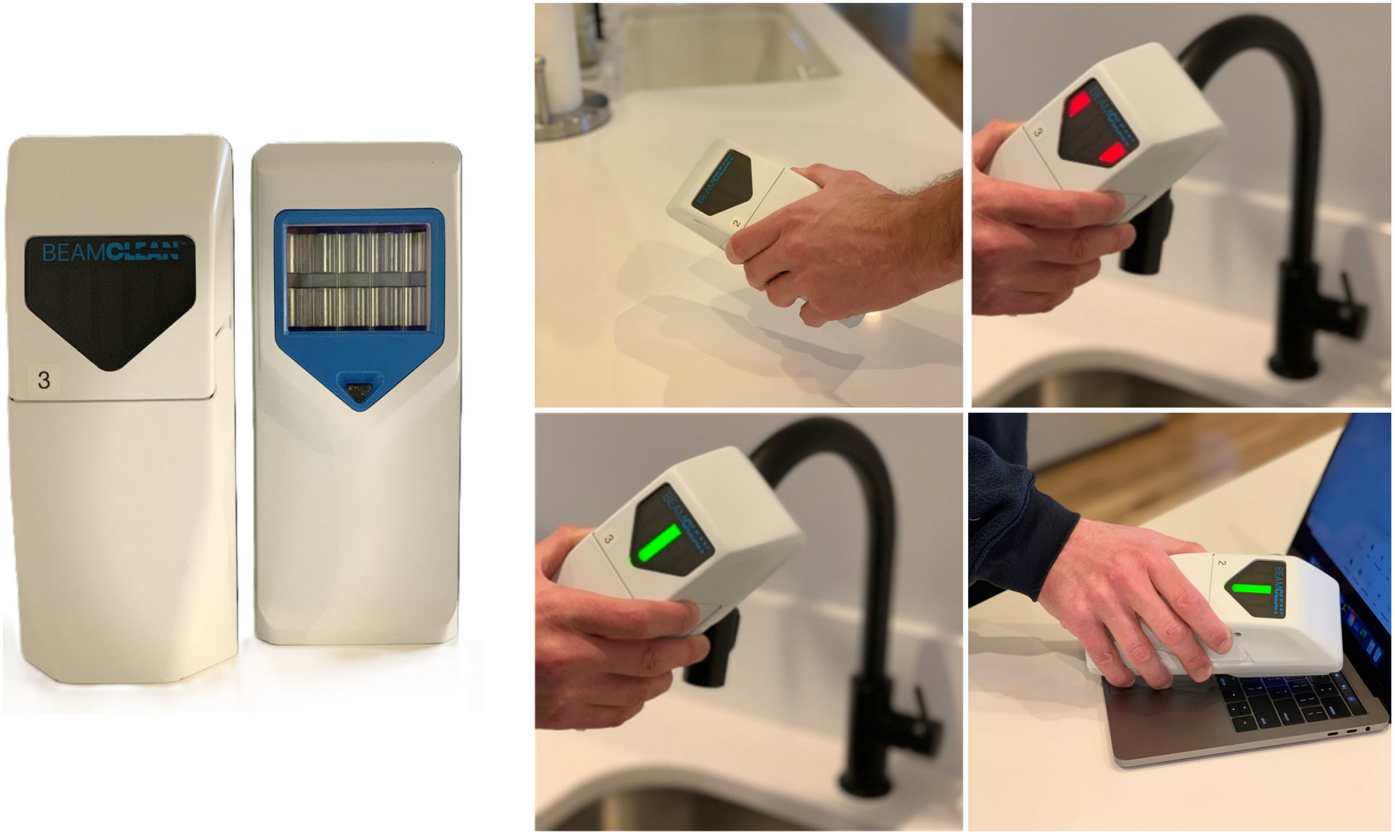
The portable filtered far UV-C handheld BeamClean FFUHH device. To treat the surface, the device is placed over the sampling area and the green light indicates that the device is at the correct distance for proper function.

Before each use, the device’s irradiance output was verified following the manufacturer’s quality control procedures. Surfaces were treated by placing the device over the sampling area at 2.5 cm or closer, with LED lights helping to guide its placement. The surface was treated for 20 seconds continuously, and then for an additional 20 seconds with small, slow circular motions to accommodate irregular and 3-dimensional surfaces (Figure 1).

### Sample collection

**Figure 2.**
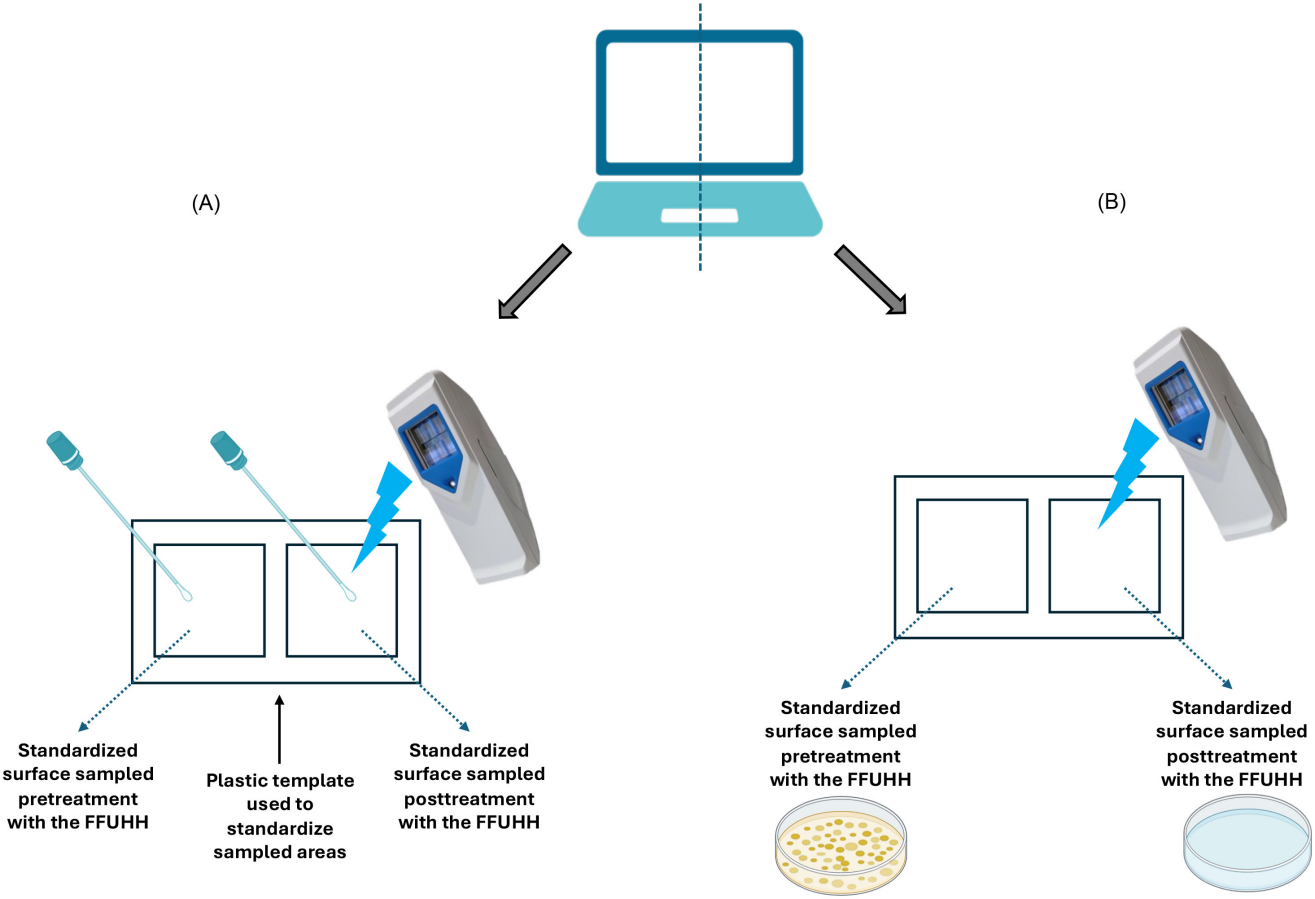
Sequential sample collection process of high-touch surfaces. For each surface, swabs (A) and contact plates (B) were collected on standardized adjacent areas. FFUHH treatment was conducted in accordance with the manufacturer’s instructions for use on an adjacent area of the high-touch surface. Posttreatment swabs and contact plates were collected immediately following FFUHH use. A plastic template delineating the areas to be sampled was used across all sample collections and sterilized before each use.

Samples were collected weekly over 24 weeks on a rotating schedule from multiple workrooms on hematologic malignancy and stem cell transplant units. Each time, the sampled items were dictation device, mouse, chair arm rests, desk surface around the keyboard, and keyboard. Four samples per surface were collected: one Tryptic Soy Agar contact plate and one Phosphate-Buffered Saline (PBS)-moistened sterile cotton swab, each before and after FFUHH treatment. For each surface item, samples were collected from standardized adjacent areas, both before and after treatment, as illustrated in Figure 2. These environmental samples are labeled “Env” for subsequent analyses.

Clinical isolates were recovered from patients admitted to the same units within 2 weeks of environmental sampling.

Finally, 5 samples for each target pathogen (designated below as “IC”) were randomly selected from our institution’s Infection Control pathogens bank, collected and stored from hospitalized patients between March 2021 and June 2023.

### Cell culture and isolation

Contact plates were incubated in aerobic conditions for 48 hours at 37±1 °C, and colony-forming units (CFUs) were counted. Swabs were enriched in Tryptic Soy Broth for 48–72 hours at 37±2 °C in aerobic conditions and then cultured on blood and MacConkey agar, along with 5 selective media: Hicrome MRSA agar, Hicrome Klebsiella selective agar (HiMedia Laboratories, USA), CHROMagar Pseudomonas, CHROMagar VRE (CHROMagar, France), and MacConkey Sorbitol ChromoSelect Agar (MilliporeSigma, USA). All media were prepared and plated per manufacturer’s instructions. The plates were then incubated for an additional 48h at 37±1 °C.

### MALDI-TOF mass spectrometry

Both clinical and environmental isolates were prepared from their respective glycerol stocks for Matrix-Assisted Laser Desorption Ionization Time-of-Flight (MALDI-TOF) mass spectrometry analysis using bioMérieux (France) and Bruker (USA) platforms. A known American Type Culture Collection (ATCC) bacterial sample or Bacterial Test Standard was loaded in the MALDI slide for determining the accuracy of bacterial identifications. Confidence levels for microbial identifications were designated by each MALDI-TOF platform. For the direct plating procedure, a single colony of bacteria was spotted directly on to the MALDI slide and overlaid with matrix solution. For samples that could not be identified via the direct plating procedure, a preparatory extraction procedure was followed, either using formic acid only or a mixture of formic acid and acetonitrile for microbial identification.

### Whole genome sequencing (WGS)

Genomic DNA was extracted using the QIAamp DNA Micro Kit (Qiagen, Germany). DNA concentration and purity were measured using the Qubit 2.0 Fluorometer (Life Technologies, USA) or NanoDrop Spectrophotometer (ThermoFisher, USA). DNA libraries were prepared using the Nextera DNA Flex Library Prep Kit (Illumina, USA) and sequenced on the Illumina NextSeq 550 platform, generating 150-bp paired-end reads.

Trimmomatic-v0.39 was used to trim low-quality base pairs and adapters from paired-end 150-bp reads. Trimmed reads underwent quality control using fastqc-v0.12.0 (i.e., Phred read quality score > 30; low adapter content). Paired-end reads were used as input to the SPAdes assembler pipeline Shovill-v1.1.0 (GitHub: Seemann, https://github.com/tseemann/shovill). Draft assemblies were quality controlled with quast-v5.2.0 (i.e., contigs <500 bp removed) and annotated using the prokka-v1.14.5 pipeline. Whole-genome multilocus sequence typing (wgMLST; assembly-free and assembly-based calls) and whole-genome single nucleotide polymorphism (wgSNP) analysis were performed using the calculation engine on the Bionumerics version 7.6 platform. *In silico* MLST was executed on the draft assemblies using the mlst-v2.22.0 command-line tool with the PubMLST database (accessed 2024-04-16). Prokka output was subsequently used to detect antimicrobial resistance (AMR) determinants and virulence factors with the NCBI AMRFinderPlus tool using the Reference Gene Database (2024-01-31.1).

### Statistical analysis

CFUs were counted on the contact plates for each surface and treatment condition. Mean reduction percentages were calculated by comparing pre- and posttreatment values for each surface. *P* values were calculated using the Wilcoxon matched pairs signed rank test and *P* values < 0.05 were considered statistically significant.

## Results

### Efficacy of the FFUHH

**Figure 3.**
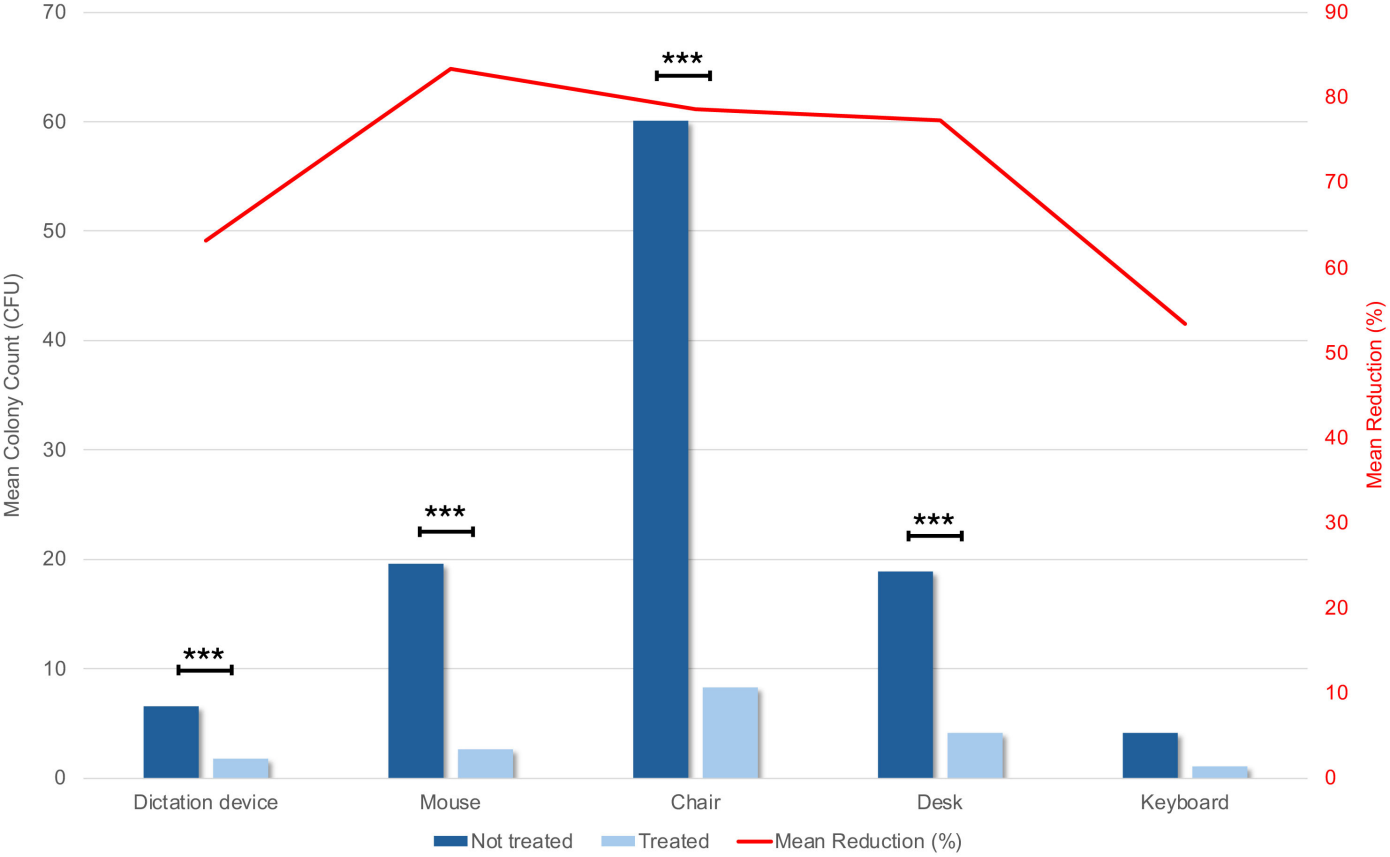
Efficacy of the UV treatment. Columns indicate mean CFUs before and after treatment with the FFUHH device for each tested surface. Mean reduction percentages were calculated by comparing pre- and posttreatment values for each surface, respectively. Statistical analysis was performed, and *P* values calculated using Wilcoxon matched pairs signed rank test. (***) indicate *P* value < 0.0001. The red line indicates the mean reduction percentage for every sampled surface.

A total of 440 contact plates were collected over the study period: 220 collected before and 220 after FFUHH treatment. The highest pretreatment mean CFU count was observed on the armrests and the lowest on keyboard. The mean percentage reduction in CFUs from pre- to posttreatment ranged from 53% for the keyboard and 83% for the mouse (Table 1 and Figure 3). The reduction was statistically significant across all surfaces except keyboards.

**Table 1.** Efficacy of the UV-C treatment. A total of 44 sampling events for each surface were considered for the statistical analysis. (***) indicates statistically significant differences (P value < 0.05; Wilcoxon matched-pairs signed-rank test). CFU, colony-forming unit; SEM, standard error of the mean

		Mean CFU ± SEM	Median CFU	95% Confidence interval	Mean % reduction ± SEM	*P* value
**Dictation Device**	**Pretreatment**	6.52 ± 1.75	3.00	3.13−9.91	63.20 ± 7.73	0.0001***
	**Posttreatment**	1.80 ± 0.46	1.00	0.91−2.68		
**Mouse**	**Pretreatment**	19.59 ± 4.083	9.00	11.68−27.50	83.35 ± 7.00	0.0001***
	**Posttreatment**	2.66 ± 1.14	0.00	0.44−4.87		
**Armchair**	**Pretreatment**	60.05 ± 20.00	24.00	21.29−98.8	78.62 ± 3.65	0.0001***
	**Posttreatment**	8.27 ± 2.72	3.50	3−13.55		
**Desk**	**Pretreatment**	18.91 ± 8.90	4.00	1.66−36.16	77.31 ± 4.78	0.0001***
	**Posttreatment**	4.14 ± 2.67	1.00	−1.04−9.31		
**Keyboard**	**Pretreatment**	4.14 ± 1.16	1.00	1.88−6.39	53.41 ± 10.37	0.371
	**Posttreatment**	1.07 ± 0.19	1.00	0.7−1.43		

### Clinically significant isolated pathogens

We isolated 40 pathogens before treatment and 28 pathogens after treatment with the FFUHH. Organisms identified by MALDI-TOF included several pathogens of the human skin microbiota, such as *Micrococcus luteus*, *Staphylococcus (S.) capitis,* as well as methicillin-resistant *S. epidermidis* (MRSE), *S. haemolyticus*, *S. ureilyticus*, and *S. hominis.* We also identified several *Candida parapsilosis*, *P. stutzeri* strains, as well as one strain each of *K. pneumoniae*, *Listeria grayi*, and *Acinetobacter (A.) baumannii*.

### Characteristics of the isolated pathogens

We identified several multidrug-resistant *S. epidermidis* ST5 strains; wgSNP analysis revealed that these strains clustered with historic linezolid-resistant ST5 strains isolated in our hospital between 2013 and 2015.^19^ However, no close genetic relationship was established between our isolates and these previously collected clinical samples.

We also identified several *S. epidermidis* ST16 strains detected on surfaces both before and after FFUHH treatment. In fact, MRSE isolates Env52 and Env58, which were collected from the same keyboard surface, post- and pretreatment, respectively, had an identical core gene alignment and differed by only one SNP. A genetically related Env57 was isolated from the mouse of the same workstation and was shown to have 4 and 5 SNPs differing from Env52 and Env58, respectively.

The wgSNP analysis of a *K. pneumoniae* isolated from a mouse surface (Env70) and a clinical carbapenem-resistant *K. pneumoniae* from the infection control bank (IC18) established a genetic relationship between the 2 isolates, which differed by 5 SNPs. Both isolates bore a rare sequence type, ST3687, that had not been previously detected in our institution. The clinical isolate was collected on a different floor, 4 months after the environmental sample collection.

### AMR characterization

All *S. epidermidis* ST16 strains were isolated on VRE-selective media, suggesting that these strains are vancomycin resistant. In addition, WGS analysis revealed that the isolates harbored multiple genes predicting resistance to multiple antibiotic classes. Specifically, isolates Env16, Env52, Env57, and Env58 all harbored resistance genes to macrolides (*mph(C)* and *msr(A)*), methicillin (*mecA and mecR1*), and β-lactams (*blaZ*, *blaR1*, and *blaI*). Interestingly, they all also harbored stress-resistance elements, in particular resistance to the biocide quaternary ammonium (*qacA* and *qacR*) (Table 2).

**Table 2.** AMR determinants and virulence elements of the environmental isolates. (*) Phenotypic observation based on the capacity of the isolates to grow on a selective media supplemented with 60 mg/L of vancomycin or 4 mg/L of methicillin. (**) Phenotype based on laboratory susceptibility testing

	Vancomycin*	*B-lactam*	*Methicillin*	*Macrolides*	*Fosfomycin*	*Fusidic acid*	*Rifamycin*	*Carbapenem*	*Quinolone*	*Quaternary ammonium*	*Heat*
*Staphylococcus epidermidis ST16*											
Env16	Yes	*blaZ, blaR1, blaI*	*mecA, mecR1*	*mph(C), mph(A)*	–	–	–	–	–	*qacA, qacR*	–
Env52	Yes	*blaZ, blaR1, blaI*	*mecA, mecR2*	*mph(C), mph(A)*	–	–	–	–	–	*qacA, qacR*	–
Env57	Yes	*blaZ, blaR1, blaI*	*mecA, mecR3*	*mph(C), mph(A)*	–	–	–	–	–	*qacA, qacR*	–
Env58	Yes	*blaZ, blaR1, blaI*	*mecA, mecR4*	*mph(C), mph(A)*	–	–	–	–	–	*qacA, qacR*	–
*Staphylococcus epidermidis ST5*											
Env05	–	*blaZ, blaR1, blaI*	Yes (*), mecA	–	*fosB*	–	–	–	–	*qacA*	–
Env44	Yes	*blaZ, blaR1, blaI*	–	*msr(A), mph(C)*	*fosB*	*fusB*	–	–	–	*qacA*	–
*Staphylococcus haemolyticus*											
Env37	Yes	–	–	*msr(A), mph(C)*	–	–	–	–	–	*qacA, qacR*	–
Env40	Yes	*blaZ, blaR1, blaI*	*mecA, mecR1, mecI*	*msr(A), mph(C)*	–	*fusb*	*arr*	–	–	*qacA, qacC, qacR*	–
*Staphylococcus ureilyticus*											
Env49	Yes	*blaZ, blaR1, blaI*	–	*msr(A), mph(C)*	–	*fusf*	–	–	–	*qacG*	–
*Klebsiella pneumoniae*											
IC18	–	*blaSHV-11*	–	–	*fosA*	–	–	Yes (**), *blaKPC-2*	*qnrS1, oqxA10, oqxB19*	–	*hdeD-GI*, *trxLHR*, *kefB-GI*, *psi-GI*, *hsp20*, *clpK*, *shsP*, *yfdX1*, *yfdX2*
Env70	–	*blaSHV-11*	–	–	*fosA*	–	–	*blaKPC-2*	*qnrS1, oqxA10, oqxB19*	–	*hdeD-GI, trxLHR, kefB-GI* *psi-GI, hsp20* *clpK, shsP, yfdX1, yfdX2*
*Acinetobacter baumannii*											
Env09	–	–	–	–	–	–	–	*bla*_*OXA-64*_	–	–	*clpK, shsP, yfdX1, yfdX2, hdeD-GI, trxLHR*

Samples Env05 and Env44, both identified as *S. epidermidis* ST5 strains by MALDI-TOF and wgSNP analyses, were confirmed to be methicillin resistant by WGS that showed the presence of the *mecA* gene. Env44 was selected on VRE-selective media, suggesting that it is vancomycin resistant without the identification of resistance genes. In addition, resistances were also predicted to quaternary ammonium (*qacA*) and to other antibiotic classes such as β-lactam (*blaZ*, *blaR1,* and *blaI*) and Fosfomycin (*fosB*) for both Env05 and Env44, and fusidic acid (*fusB*) for Env44 (Table 2).

Similarly, we isolated other Staphylococcus species, namely *S. haemolyticus* and *S. ureilyticus*, all selected on VRE-selective media, suggesting that they are vancomycin-resistant, but no resistance genes were identified. The *S. haemolyticus* Env40, and the *S. ureilyticus* Env49 both harbored resistance genes to fusidic acid (*fusb* and *fusf,* respectively), β-lactam (*blaZ*, *blaR1,* and *blaI*), and quaternary ammonium (*qacA, qacC,* and *qacR* for Env 40 and *qacG* for Env49). The *qacA* and *qacR* genes were also present in the genome of another *S. haemolyticus* isolate, Env37. Methicillin resistance genes (*mecA*, *mecR1,* and *mecI*) were detected in the genome of Env 40, and a rifamycin resistance gene (*arr*) was detected in Env49 (Table 2).

AMR determinants analysis of both *K. pneumoniae*, Env70 and IC18, confirmed the presence of the carbapenem resistance gene *blaKPC-2* in both sequences, and detected resistance genes to β-lactam (*blaSHV-11*), fosfomycin (*fosA*), and quinolone (*qnrS1, oqxA10,* and *oqxB19*) as well as several heat resistance genes (*hdeD-GI, trxLHR, kefB-GI, psi-GI, hsp20, clpK, shsP, yfdX1,* and *yfdX2*) (Table 2).

Finally, analysis of the *A. baumannii* isolate (Env09) identified the presence of a carbapenem resistance gene, *bla*_*OXA-64*_, and several heat resistance genes, *clpK*, *shsP*, *yfdX1*, *yfdX2*, *hdeD-GI*, and *trxLHR*.

## Discussion

Antimicrobial resistance is a major global public health threat.^20^ Six pathogens are identified as the leading causes of MDRO-associated deaths (*E. coli*, *S. aureus*, *K. pneumoniae*, *Staphylococcus pneumoniae*, *A. baumannii*, and *P. aeruginosa)*,^20,21^ overlapping with pathogens known to cause drug-resistant HAIs (*Enterococcus spp*, *S. aureus*, *K. pneumoniae*, *A. baumannii*, *P. aeruginosa*, *Enterobacter spp*, and *E. coli)*.^22^ These pathogens have developed AMR mechanisms that make them challenging to manage, especially in immunocompromised patient populations, thus emphasizing the importance of effective surface disinfection to prevent their transmission within the healthcare environment.

In our study, the FFUHH demonstrated efficacy in reducing the microbial burden on most studied surfaces in patient-adjacent, shared clinical areas. The mean reduction ranged from 53% for the keyboard and 83% for the mouse. This variability in efficacy may be due to technical limitations such as ergonomic limits of contact plates, which may not be an optimal choice for sample collection from irregular surfaces like keyboards, and the uniformity of treatment of these surfaces with the device. This suggests that complex, 3-dimensional surfaces may require adjustment in time or technique to achieve effective treatment. These results are consistent with previously published data evaluating the device and its disinfection efficacy both *in vitro* ^15,23^ and real-life setting.^24^ A previous study comparing the efficacy of the FFUHH to manual disinfection with sodium hypochlorite concluded that both methods had a comparable significant effect on reducing microbial burden on surfaces; however, manual methods were slightly better.^24^

Healthcare environment cleaning methods are limited by the thoroughness of hospital personnel, which has been shown to be inconsistent.^12,25^ At our institution, environmental cleaning measures for patients on isolation precautions include increased frequency of surface cleaning and use of pulse UV light-emitting devices (Xenex, USA) for terminal cleaning. However, due to safety concerns, these devices are used exclusively in closed empty rooms and are not recommended in open clinical areas. Furthermore, workspaces with computer and electronic equipment are dry dusted only and do not undergo routine surface disinfection like other clinical areas but are rather cleaned at the discretion of clinical staff. In its current form, the use of the FFUHH device is still dependent on the willingness of the staff to use it. However, far UV-C has been proven safe in preclinical and clinical studies, which offers the possibility of automated full room irradiation of occupied spaces with no additional risk for occupants as demonstrated by Sugihara and collaborators.^26^ In consequence, implementing automated irradiation of these workrooms by fixed lamps mounted on walls and directed to irradiate designated surfaces with meticulously controlled doses of UV-C might render disinfection independent from staff intervention. The FFUHH may therefore offer the advantage of providing a safe and effective means of contactless cleaning of high-touch surfaces in these areas.

Additionally, the FFUHH may overcome some of the limitations of manual methods, such as the incompatibility of liquid disinfectants with sensitive equipment. Interestingly, all analyzed environmental isolates, except *A. baumannii,* presented with a resistance genotype for quaternary ammonium (Table 2), a broad-spectrum biocide, frequently used in healthcare settings. However, concerns have emerged in recent years regarding its intense and prolonged use and the consequences for bacterial resistance and cross-resistance, thereby allowing microorganisms to persist on surfaces.^27,28^ This would explain their presence on the surfaces we sampled for this study, as use of biocides is recommended for surface disinfection at our institution. In this context, the FFUHH technology can offer an advantage for surface disinfection to eliminate biocide-resistant MDROs and potentially circumvent their persistence on surfaces.^29,30^

The MALDI-TOF analysis of the environmental isolates showed that the selective media were suboptimal in isolating the pathogens of interest from environmental samples. The isolated microorganisms were different from the clinical isolates recovered from our patients that we routinely collect all year around and store in our Infection Control laboratory as part of our standard processes (i.e. *MRSA*, *VRE*, *P. aeruginosa*, *K. pneumoniae*, and *E. coli*) which precluded us from determining if any genetic relationship between the environmental and stored clinical isolates existed.

Interestingly, a recovered *K. pneumoniae* isolate was genetically related to a carbapenem-resistant *K. pneumoniae* collected from a hospitalized patient 4 months after the environmental sampling. Transmission between elements of the clinical environment, such as contaminated surfaces, medical equipment, or the hands of healthcare workers, and the patient may have occurred. On the other hand, several studies have highlighted the scarce environmental contamination of extended-spectrum beta-lactamase (ESBL)-producing *Enterobacteriaceae*, in particular *K. pneumoniae* and *E. coli*.^31–34^ These pathogens do not survive well in the environment, including on surfaces in rooms and bathrooms of infected patients,^34^ and a prior infected occupant was not a predictor of hospital-acquired ESBL-producing gram-negative bacilli.^33^

*A. baumannii* was isolated on the same floor but different unit as the *K pneumoniae* above within a 1-month interval. The *A. baumannii* genome harbored resistance genes to carbapenem (*bla*_OXA-64_) and heat, but confirmation by susceptibility testing was not conducted.

Most of the environmental isolates were Staphylococcus species, with 23 of 68 being *S. epidermidis*. Twenty were able to grow in vancomycin-containing media, suggesting they are resistant to vancomycin. Genomic analysis also identified multiple resistance genes, mainly to methicillin, β-lactams, and macrolides but not to vancomycin (Table 2). A previous study from our institution demonstrated a link between multidrug-resistant *S. epidermidis* and patients with bacteremia with the same pathogen and showed that ST5 and ST16 were responsible for more complex infections in our immunocompromised patients.^19^

Multidrug-resistant *S. epidermidis* has emerged in recent years as a significant nosocomial pathogen, along with other coagulase-negative staphylococci (CoNS) such as *S. haemolyticus* and *S. ureilyticus*, which have become common culprits for surgical site and central-line-associated bloodstream infections.^35,36^ All isolated CoNS were resistant to methicillin and/or vancomycin based on genotypic determinants and phenotypic observations, respectively (Table 2). Interestingly, AMR analysis did not reveal any vancomycin resistance genes, but heteroresistance to this specific antibiotic has been well described.^37^ It is characterized by growth within the intermediate susceptibility range to the antibiotic while still testing as susceptible by standard experimental methods. Reduced vancomycin susceptibility could pose a serious concern, especially for immunocompromised patients, as vancomycin has long been the preferred treatment for drug-resistant gram-positive organisms.^38,39^

One of the major limitations of our study was the suboptimal performance of the selective media used to isolate pathogens, underscoring the need for improved culturing methods or strategies for recovery of pathogens from complex environmental samples. Resource constraints also restricted us to analyze 1 or 2 environmental isolates per collected sample, which may have limited our ability to identify clinically relevant minority pathogens. Additionally, although high-touch surfaces in shared clinical workrooms are not subjected to routine disinfection, they may still be cleaned by clinical staff with chemical disinfectants, at their discretion. This variability in cleaning practices may have affected our sample collection and reduced the microbial yield. Finally, the prolonged antimicrobial effects of the FFUHH after surface treatment and assessment of any residual or cumulative effects were not investigated in this study and remain unknown. However, this limitation may be overcome by recommending design changes to the manufacturer to potentially increase the surface of the treated area and even consider automation.

In summary, we demonstrated that the FFUHH efficiently reduced microorganisms on high-touch surfaces in clinical workrooms, and that its efficiency was surface-dependent. Testing the FFUHH technology on a bigger scale and on larger surfaces would prove useful to better fit the needs and the practicality of use of the device in a hospital setting. In addition, we identified several MDROs in the clinical environment, mainly multidrug-resistant staphylococci, emerging in recent years as responsible for serious nosocomial infections.
